# Author Correction: Variants in exons 5 and 6 of *ACTB* cause syndromic thrombocytopenia

**DOI:** 10.1038/s41467-018-07404-6

**Published:** 2018-11-19

**Authors:** Sharissa L. Latham, Nadja Ehmke, Patrick Y. A. Reinke, Manuel H. Taft, Dorothee Eicke, Theresia Reindl, Werner Stenzel, Michael J. Lyons, Michael J. Friez, Jennifer A. Lee, Ramona Hecker, Michael C. Frühwald, Kerstin Becker, Teresa M. Neuhann, Denise Horn, Evelin Schrock, Indra Niehaus, Katharina Sarnow, Konrad Grützmann, Luzie Gawehn, Barbara Klink, Andreas Rump, Christine Chaponnier, Constanca Figueiredo, Ralf Knöfler, Dietmar J. Manstein, Nataliya Di Donato

**Affiliations:** 1 Institute for Biophysical Chemistry, Hannover Medical School, 30625 Hannover, Germany; 2 Institute of Medical and Human Genetics, Charité-Universitätsmedizin Berlin, 13353 Berlin, Germany; 3 Berlin Institute of Health, 10117 Berlin, Germany; 4 Division for Structural Biochemistry, Hannover Medical School, 30625 Hannover, Germany; 5 Institute for Transfusion Medicine, Hannover Medical School, 30625 Hannover, Germany; 6 Department of Neuropathology, Charité-Universitätsmedizin Berlin, 10117 Berlin, Germany; 7 Greenwood Genetic Center, Greenwood, SC 29646 USA; 8 Institute for Clinical Chemistry and Laboratory Medicine, Medical Faculty of TU Dresden, 01307 Dresden, Germany; 9 Swabian Children’s Cancer Center, Children’s Hospital Augsburg, 86156 Augsburg, Germany; 10 Medical Genetics Center, 80335 Munich, Germany; 11 Institute for Clinical Genetics, TU Dresden, 01307 Dresden, Germany; 12 Core Unit for Molecular Tumor Diagnostics, National Center for Tumor Diseases Dresden, 01307 Dresden, Germany; 13 Department of Pathology-Immunology, Faculty of Medicine, University of Geneva, 1211 Geneva, Switzerland; 14 Department of Paediatric Haemostaseology, Medical Faculty of TU Dresden, 01307 Dresden, Germany

Correction to: *Nature Communications* 10.1038/s41467-018-06713-0; published online 12 Oct 2018

The original version of this Article contained an error in Fig. 4. In panel i, the lower CYA and α-SMA images were inadvertently inverted. This has been corrected in both the PDF and HTML versions of the Article.

